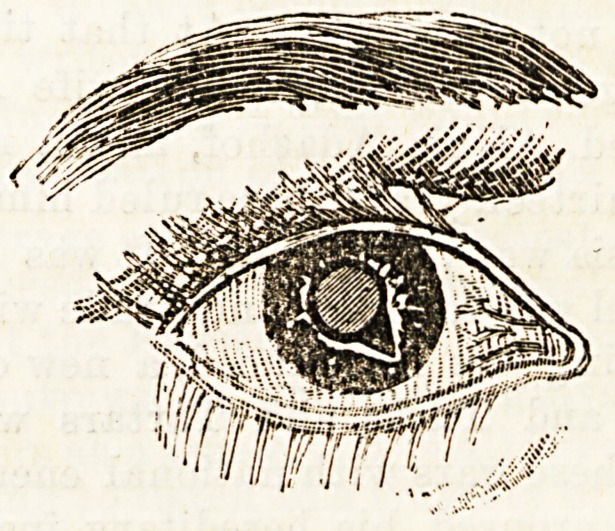# On Modern Progress in Ophthalmic Medicine and Surgery

**Published:** 1894-08-04

**Authors:** Robert Brudenell Carter

**Affiliations:** Consulting Ophthalmic Surgeon to St. George's Hospital


### On Modern Progress in Ophthalmic Medicine and Surgery.

By Robert Brudenell Carter, F.R.C.S., Consulting Ophthalmic Surgeon to St. George's Hospital.

LAMINAR CATARACT—*Continued.*

The practical failure of the late Mr. Critchett's operation of iriddesis served to turn the attention of surgeons to other methods by which an artificial pupil of somewhat similar shape and character might be obtained, and Sir William Bowman was, I believe, the first to make a practical suggestion for this purpose. He used an ordinary broad cutting needle, and a special knife something like a tiny spatula, having one cutting edge, a blunt back, and a smooth and rounded extremity. The cutting needle was made to enter the cornea near its margin, at a point opposite to the intended position of the new pupil, and was withdrawn with as little loss of aqueous humour as possible. The special knife was introduced through the resulting opening, was carried across the anterior chamber with its flat towards the lens, and was made to pass well under the iris at the side opposite to its entrance. Its sharp edge was then turned forwards and was made to cut a slit in the iris against the inner surface of the cornea. The slit at once gaped, and gaped decidedly when the aqueous humour was re-secreted, leaving a pupil of the shape desired. In this proceeding it was not difficult for a novice either to wound the lens, producing traumatic cataract, or to dislocate it; while it sometimes befell that the little knife not only cut through the iris, but also into the substance of the cornea, so that the area in front of the new pupil was bisected by a linear scar, which materially interfered with the quality of the visual result. In order to avoid this corneal scar, Dr. de Wecker performed a somewhat similar operation with a special pair of scissors constructed for him by Luer. The blunt-ended closed blades were introduced through an incision made precisely as for Bowman's operation, were opened in the anterior chamber sufficiently to allow one of them to be passed below the iris, and the other above it, were closed, and withdrawn. By this method the risk of injury to the cornea was avoided, but that of dislocating the lens was materially increased, the scissors taking up much more room than Bowman's knife. I think I may justly claim for myself to have overcome both difficulties, and to have devised a method of procedure which combines the best attainable optical results with the smallest amount of risk in the actual performance of the operation. For this purpose I make a linear incision with a very thin knife, just beyond the margin of the true cornea, and on the same side as the intended pupil. The incision should be of sufficient length to allow Luer's scissors an easy way into the anterior chamber, as there will then be less loss of aqueous humour than if they are introduced with difficulty through a small opening. The scissors are carried in closed, the back of the blades almost in contact with the iris, and the blunt ends directed forward towards the centre of the cornea. As soon as the ends reach the margin of the pupil the blades are suffered to expand a little, more or less according to the amount of iris which it is desired to remove. As they expand a tiny fold of iris is lifted between them by the aqueous humour, and is excised as they are closed. This tiny fold will then rest on the surface of the closed blades, and will float out as they are withdrawn from the eye, if care be taken to press the back of the blades against the posterior lip of the incision, and thus to cause it to gape a little. If this manœuvre should not succeed, and if the piece excised be left within the eye, it may be cautiously withdrawn by fine forceps. The resulting artificial pupil will be such as is shown in the figure, which also shows the relation of the central cataract to the opening. The operation is one which should only be undertaken by a surgeon already practised in eye work; because an unskilled person might easily either miss the iris entirely, or wound the lens, although neither of these accidents ought to be permitted to occur. In my own first attempts, and until the knack of handling the scissors had been thoroughly acquired, I more than once missed the margin of the pupil, and cut a piece out of the iris,leaving this margin intact, so as to make an additional pupil instead of an enlarged and angular one. If this should happen, the best plan is at once to introduce a Tyrrell's hook, to carry it through the new pupil and out again through the natural one, and then to break the intervening thread of iris tissue by traction. If the traction should lead to any prolapse of iris, the portion prolapsed should be replaced by a fine spatula before the eye is closed.

The foregoing operations upon the iris are alike in this, that they are only applicable to cases in which the opaque lamina is of small diameter, so that portions of the lens, not too near its absolute margin, are still transparent and available for visual purposes. It has been said, moreover, by many observers, that in the cases in which the transparent margin, however wide, is interrupted by radiating lines of opacity, these radiating lines tend to increase and coalesce, so that complete cataract may be expected at an early period of life. I am not quite sure whether this statement is borne out by facts, or whether it is one which has passed, with strange vitality, from one text book to another, but it is at least commonly believed and acted upon. In the cases, therefore, of laminar cataract of large diameter, and in those which present radiating opacities, the choice rests between removal of the lens and submission to the defect; and this choice would manifestly be much guided by two considerations; first, the degree of ease and safety with which the removal of the lens could be effected; and secondly, the extent to which the defect was likely to interfere with the prospects or the usefulness of the subject. This would depend, obviously, partly upon the depth of the opacity, and partly upon the position of the individual. In former years, the recognised method of treating laminar cataracts, when the removal of the lens was decided upon, was by the method of absorption; but this has been very generally superseded by the operation of suction, which is said to be of Persian origin, and which was introduced into this country by Mr. Pridgin Teale. An account of the merits and defects of the two methods must be reserved for the next paper.

## Figures and Tables

**Figure f1:**